# Toward Personalized Interventions for Psoriasis Vulgaris: Molecular Subtyping of Patients by Using a Metabolomics Approach

**DOI:** 10.3389/fmolb.2022.945917

**Published:** 2022-07-19

**Authors:** Dan Dai, Chunyan He, Shuo Wang, Mei Wang, Na Guo, Ping Song

**Affiliations:** ^1^ Department of Dermatology, Guang’anmen Hospital, China Academy of Chinese Medical Sciences, Beijing, China; ^2^ Department of Dermatology, Hubei Provincial Hospital of TCM, Wuhan, China; ^3^ Department of Oncology, Guang’anmen Hospital, China Academy of Chinese Medical Sciences, Beijing, China; ^4^ Leiden University-European Center for Chinese Medicine and Natural Compounds, Institute of Biology Leiden, Leiden University, Leiden, Netherlands; ^5^ SU BioMedicine, BioPartner Center 3, Leiden, Netherlands; ^6^ Experimental Research Center, China Academy of Chinese Medical Sciences, Beijing, China

**Keywords:** psoriasis vulgaris, metabolomics, lipid metabolites, severity biomarkers, metabolic diseases, molecular subtyping

## Abstract

**Aim:** Psoriasis vulgaris (PV) is a complicated autoimmune disease characterized by erythema of the skin and a lack of available cures. PV is associated with an increased risk of metabolic syndrome and cardiovascular disease, which are both mediated by the interaction between systemic inflammation and aberrant metabolism. However, whether there are differences in the lipid metabolism between different levels of severity of PV remains elusive. Hence, we explored the molecular evidence for the subtyping of PV according to alterations in lipid metabolism using serum metabolomics, with the idea that such subtyping may contribute to the development of personalized treatment.

**Methods:** Patients with PV were recruited at a dermatology clinic and classified based on the presence of metabolic comorbidities and their Psoriasis Area and Severity Index (PASI) from January 2019 to November 2019. Age- and sex-matched healthy controls were recruited from the preventive health department of the same institution for comparison. We performed targeted metabolomic analyses of serum samples and determined the correlation between metabolite composition and PASI scores.

**Results:** A total of 123 participants, 88 patients with PV and 35 healthy subjects, were enrolled in this study. The patients with PV were assigned to a “PVM group” (PV with metabolic comorbidities) or a “PV group” (PV without metabolic comorbidities) and further subdivided into a “mild PV” (MP, PASI <10) and a “severe PV” (SP, PASI ≥10) groups. Compared with the matched healthy controls, levels of 27 metabolites in the MP subgroup and 28 metabolites in the SP subgroup were found to be altered. Among these, SM (d16:0/17:1) and SM (d19:1/20:0) were positively correlated with the PASI in the MP subgroup, while Cer (d18:1/18:0), PC (18:0/22:4), and PC (20:0/22:4) were positively correlated with the PASI in the SP subgroup. In the PVM group, levels of 17 metabolites were increased, especially ceramides and phosphatidylcholine, compared with matched patients from the PV group. In addition, the correlation analysis indicated that Cer (d18:1/18:0) and SM (d16:1/16:1) were not only correlated with PASI but also has strongly positive correlations with biochemical indicators.

**Conclusion:** The results of this study indicate that patients with PV at different severity levels have distinct metabolic profiles, and that metabolic disorders complicate the disease development. These findings will help us understand the pathological progression and establish strategies for the precision treatment of PV.

## Background

Psoriasis is a chronic, relapsing, immunoinflammatory skin disease that affects nearly 125 million of the global population (Takeshita et al., 2017). Psoriasis vulgaris (PV), also known as “plaque-type psoriasis,” accounts for approximately 90% of all cases (Boehncke and Schön, 2015) and has a multifactorial etiology, including polygenetic disorders, environmental factors, inflammation, and mental health (e.g., depression) (Kamiya et al., 2019). PV progression has been reported to be closely associated with metabolic disorders, such as obesity, diabetes, hypertension, and cardiovascular disease (Elmets et al., 2019; Kim et al., 2019), which may be attributed to the interplay between inflammation and metabolic dysfunction (Dutkiewicz et al., 2016; Kang et al., 2017). Aberrations in lipid expression and metabolism, as well as in receptors, enzymes, and lipid transport proteins, are frequently observed in patients with psoriasis. Such lipid abnormalities exacerbate psoriatic lesions and increase the risk of developing hyperlipidemia, metabolic syndrome, and cardiovascular disorders (Jia et al., 2018; Cai et al., 2021; Nowowiejska et al., 2021). Moreover, the drugs used in psoriasis therapy may have an impact on the patient’s lipid profile (Cao et al., 2021); therefore, monitoring the lipid profile is of great importance not only to disease development but also for possible adverse response caused by treatments. A high-fat diet rich in saturated fatty acids not only induces obesity but also exacerbates psoriasiform dermatitis (Herbert et al., 2018). A large clinical observation sample also highlighted the link between lipid metabolites and cardiovascular events in patients with PV (Colaco et al., 2021). The treatments of PV involve a variety of medications such as interleukins, nonsteroidal anti-inflammatory drugs, lithium, interferons, beta-blockers, antimalarial medications, calcium channel blockers, terbinafine (Jain, 2017), lipid-lowering medications (James, 2005), and contentious TNF inhibitors such as infliximab or adalimumab (Guerra and Gisbert, 2013). Untargeted metabolomics data revealed that interleukin-17A monoclonal antibody (ixekizumab) treatment improves lipids metabolism and has the potential to reduce the cardiovascular risk in patients with psoriasis (Cao et al., 2021). Therefore, developing a personalized intervention strategy using an advanced technology platform will be cost-effective and increase the quality of life of patients. For this purpose, we first aimed to stratify patients with PV into subtypes. The inherent relationship between PV and metabolic diseases sheds lights on new intervention strategies.

Metabolomics approaches enable us to decode the complex perturbations between individual genetic inheritance and dynamic environments by combining high-throughput analysis technology combined with pattern recognition and expert systems (Donnelly et al., 2019). Such methods have been applied to capture the overall metabolic trajectory of patients with PV by profiling small molecular metabolites (Kamleh et al., 2015; Alonso et al., 2016; Ottas et al., 2017). As the molecular mechanisms underlying PV pathogenesis are not fully understood, it is difficult to identify a conclusive method for PV treatment (Raychaudhuri et al., 2014). The application of metabolomics is of great importance in exploring pathological features for successful clinical management and individualized medicine (Tarentini et al., 2021). Kang et al. (2017) identified hypoxanthine, ornithine, azelaic acid, and crotonic acid as candidate biomarkers for patients with psoriasis in an untargeted serum metabolomics study. Kamleh et al. (2015) revealed that PV has specific metabolic profiles at different degrees of severity, and that the severity-associated metabolic perturbations may stem from keratinocyte hyperproliferation, active collagen synthesis, or the incidence of cachexia. However, whether differences in lipid metabolism can indeed be observed in different severities of PV remains largely unknown.

Here, we therefore recruited patients with PV as well as healthy controls (HCs) and further subclassified the patients by the presence of metabolic comorbidities and their Psoriasis Area and Severity Index (PASI). A targeted lipidomics approach based on ultra-performance liquid chromatography–tandem mass spectrometry (UPLC-MS/MS) was applied to characterize lipid biomarkers for PV subtyping and examine distinct metabolic signatures.

## Materials and Methods

### Participant Selection

Patients with PV and HC participants were recruited at Guang’anmen Hospital, China Academy of Chinese Medical Sciences (Beijing, China). We posted recruitment advertisements on the hospital’s websites and notice boards, providing a brief overview of the study’s goals, the medical assessments participants were to undergo, the eligibility criteria, and instructions on how to get involved. Patients were enrolled based on the following criteria: 1) aged 18–65 years, 2) diagnosis of PV, and 3) provision of a signed informed consent form. The following participants were excluded: 1) those who underwent systemic treatment within 4 weeks; 2) those with a diagnosis of pustular psoriasis, psoriatic arthritis, or erythrodermic psoriasis; 3) those with severe cardiovascular or cerebrovascular disease, abnormal liver or renal function, cancer, or psychosis disorders; and 4) pregnant or lactating women. None of the participants had dietary restrictions. Enrolled patients with PV were assigned to either the “PVM group” (PV with metabolic diseases) or the “PV group” (PV without metabolic diseases) and further subdivided into the “mild PV” (MP, PASI <10) and the “severe PV” (SP, PASI ≥10) groups. Healthy individuals without a history of psoriasis, chronic inflammatory systemic diseases, or obesity-related metabolic diseases served as the HC group and were age- and sex-matched to the MP and SP subgroups. Participants’ eligibility was examined by two dermatology physicians at the dermatological clinic of our institution, both deemed them fit for this study. Written informed consent was obtained from all participants. Serum samples were collected and analyzed from each recruited participant to obtain lipid biomarkers for PV subtyping. The recruitment process began in January 2019 and was completed in November 2019.

### Chemicals and Materials

We purchased internal standards for ceramide (Cer (d18:1/17:0)), phosphatidylethanolamine (PE [12:0/13:0]), sphingomyelin (SM [d18:1/12:0]), and phosphatidylcholine (PC (19:0/19:0)) from Avanti Polar Lipids (Alabaster, AL, United States). Free fatty acid (FFA C19:0) was sourced from Larodan (Stockholm, Sweden). MS-grade isopropanol, acetonitrile, methanol, and formic acid were purchased from Fisher Scientific (Waltham, MA, United States). Ultra-pure water was obtained from a Milli-Q water purification system and used throughout the experiments (Millipore, Bedford, MA, United States). All other reagents and chemicals used were of analytical grade and commercially available.

### Sample Preparation

Following collection, whole blood samples were centrifuged for 10 min at 3,000 rpm in a refrigerated centrifuge (4°C), after which the supernatants were aliquoted into 1.5-ml Eppendorf tubes and stored at −80°C until the subsequent metabolomic analysis. Standard stock solutions were prepared by dissolving the compounds in methanol to a concentration of 1 mg/ml and stored below −20°C.

For the targeted lipid and fatty acid analyses, 10 μl of serum was added to 150 μl of cold methanol containing a mixture of the following internal standards: FFA C19:0 (200 ng/ml), SM (d18:1/12:0) (40 ng/ml), PC (19:0/19:0) (30 ng/ml), Cer (d18:1/17:0) (200 ng/ml), and PE (12:0/13:0) (100 ng/ml). The samples were mixed for 30 s, after which 500 µl of methyl tert-butyl ether was added, and the samples were incubated under gentle agitation for 20 min at room temperature to extract the full lipids. After the addition of 125 µl of water, the samples were shaken and centrifuged for 10 min at 13,200 rpm at 4°C. The upper lipid extracts of 100 µl were transferred into a new centrifuge tube, vacuum-dried, and resuspended in a 200 µl solution of acetonitrile/isopropanol/water (65:30:5, v/v/v). The samples were again vortexed for 1 min and centrifuged as described previously, and the supernatants were instantly analyzed using UPLC-MS/MS.

To validate the stability of the LC-MS system, pooled quality control (QC) samples were prepared by mixing 1 ml of each serum sample prepared as described previously.

### UPLC-MS/MS Conditions

The UPLC and MS systems were controlled using MassLynx Mass Spectrometry Software (v4.1) (Waters Corp., MA, United States). All chromatographic separations were performed using an Acquity UPLC BEH C8 Column (1.7 μm, 100 × 2.1 mm^2^; Waters Corp.). The mobile phase consisted of 5 mM ammonium formate in acetonitrile/water (6:4, v/v; mobile phase A) and 5 mM ammonium formate in isopropanol/acetonitrile (9:1, v/v; mobile phase B). The column was maintained at 55°C, and the flow rate was set at 0.26 ml/min. The linear elution gradient settings are shown in Supplementary Table S1. An injection volume of 1 μl was applied in the positive ion mode and a 2 μl volume in the negative ion mode. During the mass spectrometer (Xevo TQ-S; Waters Corp.) operation, we applied multiple reaction monitoring in the positive and negative ion modes. The TargetLynx Application Manager (Waters Corp.) was used to process the data.

### System Stability

To guarantee the stability of the LC-MS system throughout our analyses, we evaluated the pooled QC samples in both the positive and the negative ion modes. The retention time and intensity measurements suggested that the stability and repeatability of the measurements was satisfactory and high, respectively, throughout for the experiment. The bulk of the coefficient of variation (CV) values belonging to the internal standard peak area for the targeted lipid and fatty acid tests fell below 15%.

### Data Analysis

Raw data from the UPLC-MS/MS analysis were first imported into Progenesis QI Software (v2.3; Waters Corp.). Baseline filtering, peak identification, integration, retention time correction, and peak alignment were then performed to optimize the setting parameters. All chromatographic peaks were confirmed manually by careful investigation to verify the accuracy of the results, and a data matrix was obtained, including information such as mass-to-charge ratio (m/z), retention time, and peak area (intensity). The data matrix obtained was exported to SIMCA software (v14.1; Umetrics, Umeå, Sweden) for partial least squares discriminant analysis (PLS-DA). The *R* ^2^ and *Q* ^2^ statistics were used to assess the quality of the PLS-DA model, whereas its reliability was determined using a permutation test, because of the model’s potential to overestimate the separation performance. All statistical tests were performed using SPSS (v25.0; SPSS Inc., IL, United States). The non-parametric Student’s *t*-test, Kruskal–Wallis test, and chi-square test were used to compare the data, as appropriate. The *p*-values of <0.05 were considered significant, and no corrections were performed for multiple testing as the study was exploratory. The variables of importance in projection (VIP) analysis following PLS-DA modeling and a Student’s *t*-test analysis were applied to identify biomarkers (VIP >1, *p* < 0.05). Spearman’s correlation coefficients were calculated to study the associations between the expression of metabolites, PASI, and biochemical indicators using the ggplot2 package (v3.3.5) in R (https://www.R-project.org).

## Results

### Participant Baseline Characteristics

In total, 123 participants, including 88 patients with PV and 35 HCs, were enrolled in this study. The PV group consisted of 63 patients, and the PVM group consisted of 25; the patients in the PV group were further subdivided into 31 in the MP subgroup and 32 in the SP subgroup. We performed four pairwise comparisons, one between the MP subgroup and matched HCs (MP vs. HC-MP; 31 vs. 31), one between the SP subgroup and matched HCs (SP vs. HC-SP; 32 vs. 32), one between both subgroups (MP vs. SP; 31 vs. 32), and one between the PVM group and matched patients from the PV group (PVM vs. PV, 25 vs. 25). The HC-MP and HC-SP subgroups were based on the same 25 shared healthy subjects. The characteristics of the study cohorts are shown in Table 1, and the metabolic complications of the patients in the PVM group are illustrated in Supplementary Table S2. Among the 38 patients with PV whose PASI was <10, we identified 7 (18.42%) patients with metabolic disorders, while of the 50 patients with PV whose PASI was ≥10, 18 (36%) had metabolic disorders (Table 2). These data demonstrated a significant increase in metabolic disorders in patients with severe PV, suggesting that dysfunctions in lipid metabolism may contribute to the progression of PV.

**TABLE 1:** Demographics of the study cohort.

Comparison 1			
Characteristic	MP patient (*n* = 31)	HC-MP subject (*n* = 31)	*p*-value
Age (years)	19–62, 33.45 ± 11.38	19–58, 34.84 ± 10.74	0.667
Female	14	18	0.309
PASI	6.08 ± 2.52	NA	NA
Duration of psoriasis (years)	9.81 ± 7.01	NA	NA
Family history	11	NA	NA
BMI (kg/m^2^)	23.23 ± 2.98	22.35 ± 2.66	0.383
Total cholesterol (mmol/L)	4.53 ± 0.63	4.77 ± 0.79	0.252
Triglyceride (mmol/L)	1.37 ± 0.77	0.91 ± 0.26	0.071
LDL cholesterol (mmol/L)	2.76 ± 0.43	2.94 ± 0.58	0.203
HDL cholesterol (mmol/L)	1.29 ± 0.26	1.43 ± 0.28	0.063
**Comparison 2**			
**Characteristic**	**SP patient (*n* = 32)**	**HC-SP subject (*n* = 32)**	***p*-value**
Age (years)	19–57, 36.03 ± 10.16	19–58, 36.25 ± 11.38	0.92
Female	13	19	0.134
PASI	17.69 ± 6.41	NA	NA
Duration of psoriasis (years)	13.07 ± 8.26	NA	NA
Family history	9	NA	NA
BMI (kg/m^2^)	23.33 ± 3.99	22.34 ± 2.60	0.243
Total cholesterol (mmol/L)	4.68 ± 0.90	4.79 ± 0.82	0.665
Triglyceride (mmol/L)	1.23 ± 0.56	0.94 ± 0.26	0.059
LDL cholesterol (mmol/L)	2.92 ± 0.72	2.97 ± 0.58	0.812
HDL cholesterol (mmol/L)	1.27 ± 0.30	1.41 ± 0.26	0.066
**Comparison 3**			
**Characteristic**	**MP patient (*n* = 31)**	**SP patient (*n* = 32)**	***p*-value**
Age (years)	19–62, 33.45 ± 11.38	19–57, 36.03 ± 10.16	0.346
Female	14	13	0.716
PASI	6.08 ± 2.52	17.69 ± 6.41	0
Duration of psoriasis (years)	9.81 ± 7.01	13.07 ± 8.26	0.097
Family history	11	9	0.53
BMI (kg/m^2^)	23.23 ± 2.98	23.33 ± 3.99	0.91
Total cholesterol (mmol/L)	4.53 ± 0.63	4.68 ± 0.90	0.488
Triglyceride (mmol/L)	1.37 ± 0.77	1.23 ± 0.56	0.741
LDL cholesterol (mmol/L)	2.76 ± 0.43	2.92 ± 0.72	0.339
HDL cholesterol (mmol/L)	1.29 ± 0.26	1.27 ± 0.30	0.766
**Comparison 4**			
**Characteristic**	**PVM patient (*n* = 25)**	**PV patient (*n* = 25)**	***p*-value**
Age (years)	26–65, 45.04 ± 9.34	24–57, 42.60 ± 8.22	0.332
Female	9	9	1
PASI	18.99 ± 14.62	15.62 ± 8.74	0.816
Duration of psoriasis (years)	15.83 ± 10.76	14.48 ± 8.23	0.621
Family history	7	7	1
BMI (kg/m^2^)	27.29 ± 3.75	23.91 ± 3.28	0.002
Total cholesterol (mmol/L)	5.49 ± 1.13	5.06 ± 0.77	0.165
Triglyceride (mmol/L)	2.85 ± 1.27	1.43 ± 0.64	0
LDL cholesterol (mmol/L)	3.56 ± 0.88	3.13 ± 0.64	0.084
HDL cholesterol (mmol/L)	1.15 ± 0.19	1.29 ± 0.34	0.107

BMI, body mass index; HDL-C, high-density lipoprotein cholesterol; HC-MP, healthy controls matched with mild psoriasis vulgaris; HC-SP, healthy controls matched with severe psoriasis vulgaris; LDL-C, low-density lipoprotein cholesterol; MP, mild psoriasis vulgaris (PASI <10); PASI, Psoriasis Area and Severity Index; PVM, psoriasis vulgaris with metabolic diseases; PV, psoriasis vulgaris without metabolic diseases; SP, severe psoriasis vulgaris (PASI ≥10).

**TABLE 2:** Metabolic disorder in sub-population of PV.

Patient	PASI <10	PASI ≥10
No. of PV patient	38	50
No. of PVM patient	7 (18.42%)	18 (36%)

### Targeted Metabolomic Analysis

In our targeted metabolomics analysis, we focused on the expressions of specific lipids and fatty acids in the different groups. During the UPLC-MS/MS analysis, QC samples were regularly run to ensure the reproducibility of the results, and the majority of the CV values corresponding to the internal standard peak area were <15%, which is consistent with good stability and reliability. In total, 25 FFAs and 131 lipid metabolites were detected. Relevant information on precursor ions, product ions, and collisions for all the lipid and fatty acid samples is provided in Supplementary Table S3.

### Serum Metabolic Profiling Identifies Patients With PV at Different Severity Levels

**FIGURE 1:**
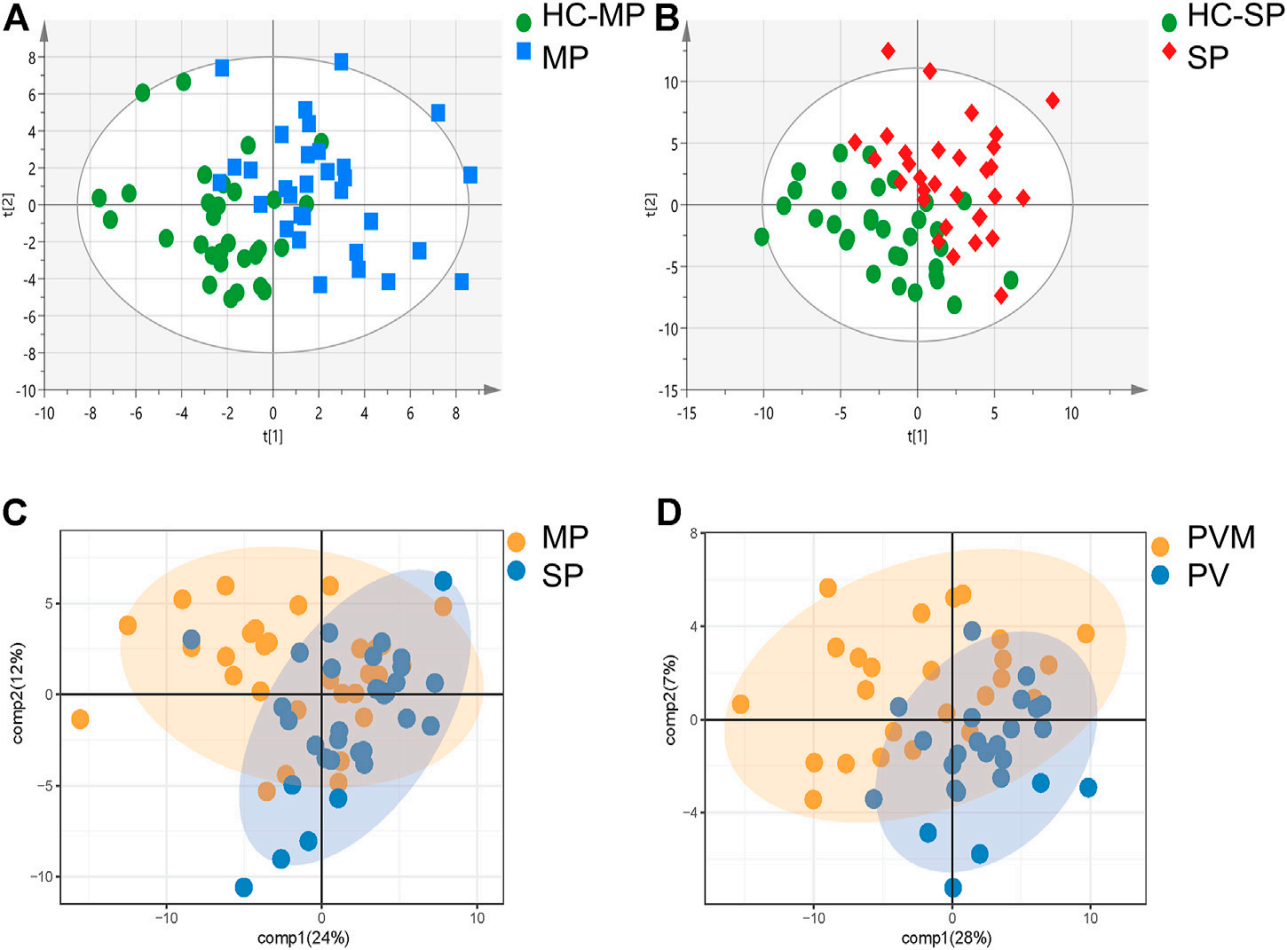
Comparison of metabolites using PLS-DA models. **(A)** PLS-DA model of the MP and HC-MP subgroups. **(B)** PLS-DA model of the SP and HC-SP subgroups. **(C)** PLS-DA model of the MP and SP subgroups. **(D)** PLS-DA model of the PVM and PV groups. HC-MP, healthy controls matched to the group with mild psoriasis vulgaris; HC-SP, healthy controls matched to the group with severe psoriasis vulgaris; MP, mild psoriasis vulgaris; PLS-DA, partial least squares discriminant analysis; PV, psoriasis vulgaris without metabolic diseases; PVM, psoriasis vulgaris with metabolic diseases; SP, severe psoriasis vulgaris.

The PLS-DA analysis was performed to investigate the metabolic differences among the groups and subgroups (Figure 1). Permutation tests (200 permutations) were performed to verify the PLS-DA models. Both the permuted *R* ^2^ and *Q* ^2^ values were significantly lower than the corresponding original values (see Supplementary Figure S1), suggesting good compatibility of the data and predictive ability of the model. Compared with the matched HC-MP and HC-SP control groups, participants in the MP and SP subgroups exhibited a significantly distinctive metabolic signature, suggesting dysfunctional lipid metabolism in patients with PV (Figures 1A,B). Although the MP subgroup coincided with the SP group, PC, LPC, and SM showed alterations between the two (Figure 1C). A clear distinction was also observed between the PVM group and matched patients in the PV group, with differences in Cer, FFA, LPC, PC, and PE between the two groups (Figure 1D). This finding suggests an overlap of PV and metabolic disorders (Table 3).

**TABLE 3:** Significantly altered lipids between different groups.

Lipid	MP vs. HC-MP	SP vs. HC-SP	SP vs. MP	PVM vs. PV
Cer	–	√	–	√
FFA	√	√	–	√
LPC	√	√	√	√
LPE	–	√	–	–
PC	√	√	√	√
PE	√	√	–	√
SM	√	√	√	–

Cer, ceramide; FFA, free fatty acid; LPC, lysophosphatidylcholine; LPE, lysophosphatidylethanolamine; PC, phosphatidylcholine; PE, phosphatidylethanolamine; SM, sphingomyelin.

### Altered Metabolites in Patients With PV at Different Severity Levels

**FIGURE 2:**
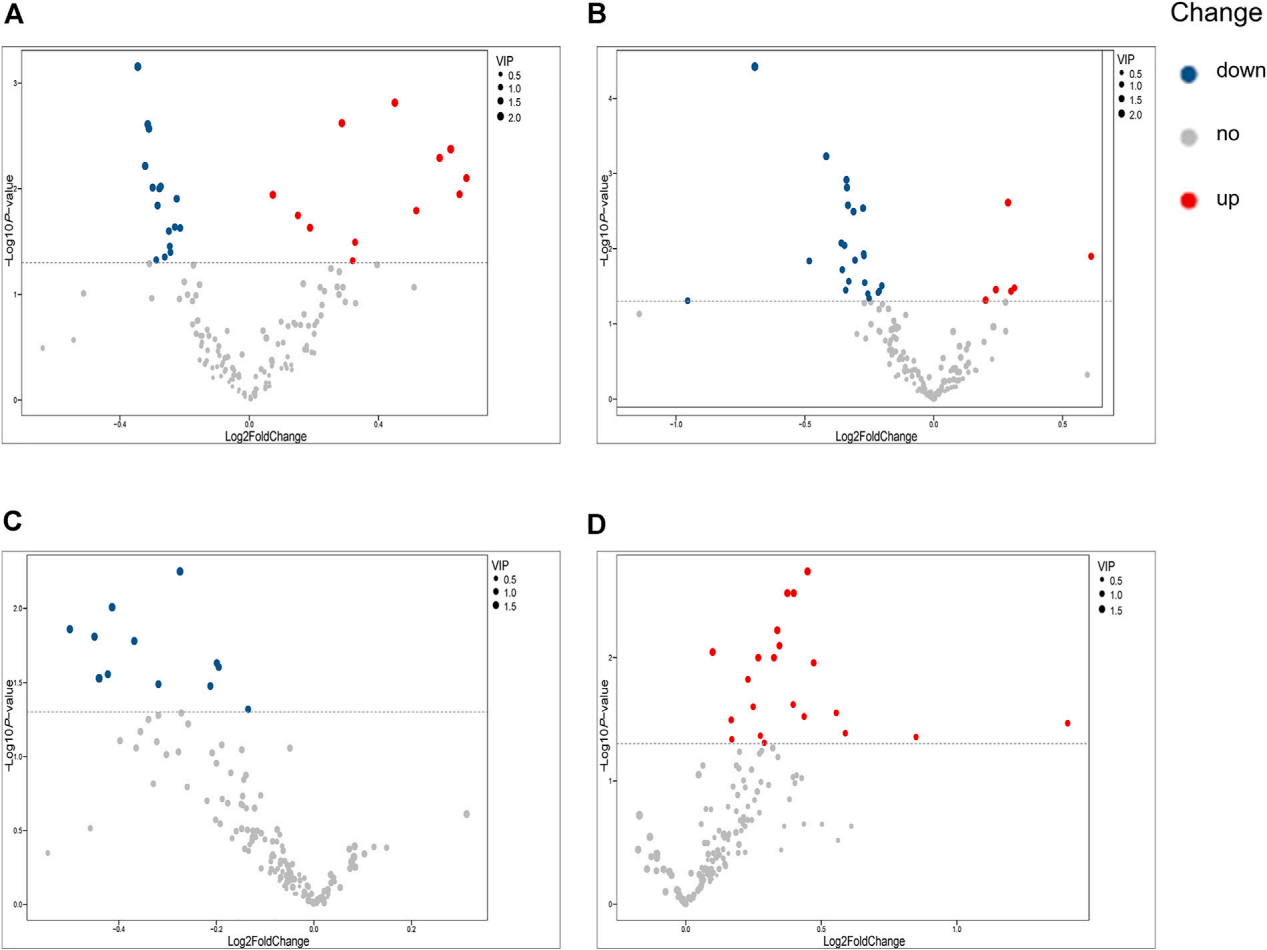
Volcano plots illustrating differential metabolites. **(A)** Volcano plot of differential metabolites between the MP and HC-MP subgroups. **(B)** Volcano plot of differential metabolites between the SP and HC-SP subgroups. **(C)** Volcano plot of differential metabolites between the MP and SP subgroups. **(D)** Volcano plot of differential metabolites between the PVM and PV groups. HC-MP, healthy controls matched to the group with mild psoriasis vulgaris; HC-SP, healthy controls matched to the group with severe psoriasis vulgaris; MP, mild psoriasis vulgaris; SP, severe psoriasis vulgaris; PV, psoriasis vulgaris without metabolic diseases; PVM, psoriasis vulgaris with metabolic diseases.

Our univariate statistical analysis revealed significant differences in lipid and fatty acid levels among the groups and subgroups (Table 4). The MP subgroup exhibited higher levels of three types of PE, six types of PC, and FA 16:2, and lower levels of five types of PC, four types of LPC, seven types of SM, and PE (18:2/16:1) than the HC-MP subgroup (Figure 2A). In the comparison between the SP and HC-SP subgroups, the levels of two types of PC, two types of Cer, PE (18:2/16:0), and FA 16:2 were elevated in the SP subgroup, while the levels of two types of LPC, ten types of SM, nine types of PC, and lysophosphatidylethanolamine 20:2 (LPE 20:2) were reduced (Figure 2B). The lower levels of PC, LPC, and SM observed in the SP subgroup distinguished it from the MP subgroup (Figure 2C). As for the comparison between the PVM and PV groups, levels of six types of Cer, two types of FFA, seven types of PC, LPC 20:2, and PE (22:5/16:0) were all significantly high in the PVM group (Figure 2D), indicating that the disorder in lipid metabolism served a critical role in metabolic complications of psoriasis.

**TABLE 4:** Expression trends of metabolites altered between different groups.

Metabolite	MP vs. HC-MP	SP vs. HC-SP	SP vs. MP	PVM vs. PV
Cer (d18:1/16:0)	–	–	–	–
Cer (d18:1/18:0)	–	△↑	–	–
Cer (d18:1/24:1)	–	△↑	–	*↑
Cer (d18:1/25:0)	–	–	–	△↑
Cer (d18:1/22:0)	–	–	–	*↑
Cer (d18:1/22:2)	–	–	–	*↑
Cer (d18:1/24:0)	–	–	–	*↑
Cer (d18:2/22:0)	–	–	–	△↑
FA 16:2	*↑	△↑	–	–
FA 19:0	–	–	–	△↑
FA 24:0	–	–	–	△↑
LPC 17:0	△↓	–	–	–
LPC 20:0	△↓	–	–	–
LPC 20:2	–	–	–	△↑
LPC 20:3	–	–	△↓	–
LPC 20:5	–	△↓	△↓	–
LPC 21:0	△↓	–	–	–
LPC 22:0	△↓	–	–	–
LPC 22:6	–	△↓	–	–
LPE 20:2	–	△↓	–	–
PC (10:0/19:1)	△↓	*↓	–	–
PC (16:1/16:0)	△↑	–	△↓	–
PC (16:0/17:1)	–	–	△↓	–
PC (14:0/18:2)	–	–	–	*↑
PC (18:0/16:0)	–	–	–	△↑
PC (15:1/18:2)	△↓	△↓	–	–
PC (16:1/18:2)	–	–	△↓	△↑
PC (18:3/16:0)	–	–	△↓	–
PC (20:4/14:0)	–	–	△↓	△↑
PC (18:1/17:0)	–	△↓	–	–
PC (18:2/17:0)	–	△↓	–	–
PC (18:2/19:1)	–	–	–	△↑
PC (17:1/18:2)	–	–	△↓	–
PC (18:0/18:1)	△↑	–	△↓	–
PC (20:4/16:1)	–	△↓	△↓	–
PC (20:4/17:0)	–	*↓	–	–
PC (15:0/22:6)	△↓	△↓	–	–
PC (18:0/20:2)	△↑	–	–	△↑
PC (18:0/20:5)	–	–	△↓	–
PC (18:0/22:4)	△↑	△↑	–	–
PC (18:0/22:5)	△↑	–	–	–
PC (20:4/20:4)	△↓	△↓	–	–
PC (20:4/22:6)	△↓	△↓	–	–
PC (20:0/18:0)	–	–	–	*↑
PC (20:0/22:4)	△↑	△↑	–	–
PE (12:0/13:0)	–	–	–	–
PE (16:0/16:0)	–	–	–	–
PE (16:0/18:1)	*↑	–	–	–
PE (18:2/16:0)	△↑	△↑	–	–
PE (18:2/16:1)	△↓	–	–	–
PE (18:0/22:5)	△↑	–	–	–
PE (22:5/16:0)	–	–	–	△↑
SM (d16:0/15:1)	–	#↓	–	–
SM (d16:1/16:0)	–	△↓	△↓	–
SM (d16:1/17:0)	*↓	*↓	–	–
SM (d18:1/15:1)	△↓	*↓	–	–
SM (d18:1/17:0)	△↓	△↓	–	–
SM (d18:1/17:1)	*↓	*↓	–	–
SM (d18:1/19:0)	–	–	–	–
SM (d18:1/19:1)	△↓	△↓	–	–
SM (d19:1/20:0)	△↓	*↓	–	–
SM (d20:0/22:6)	–	△↓	–	–
SM (d17:1/26:1)	–	△↓	–	–
SM (d16:0/22:3)	–	–	–	–
SM (d16:0/17:0)	△↓	–	–	–

△, *p* < 0.05; *, *p* < 0.01; and #, *p* < 0.001

### Associations Between the Altered Metabolites and the Metabolic Signatures of Severity

**FIGURE 3:**
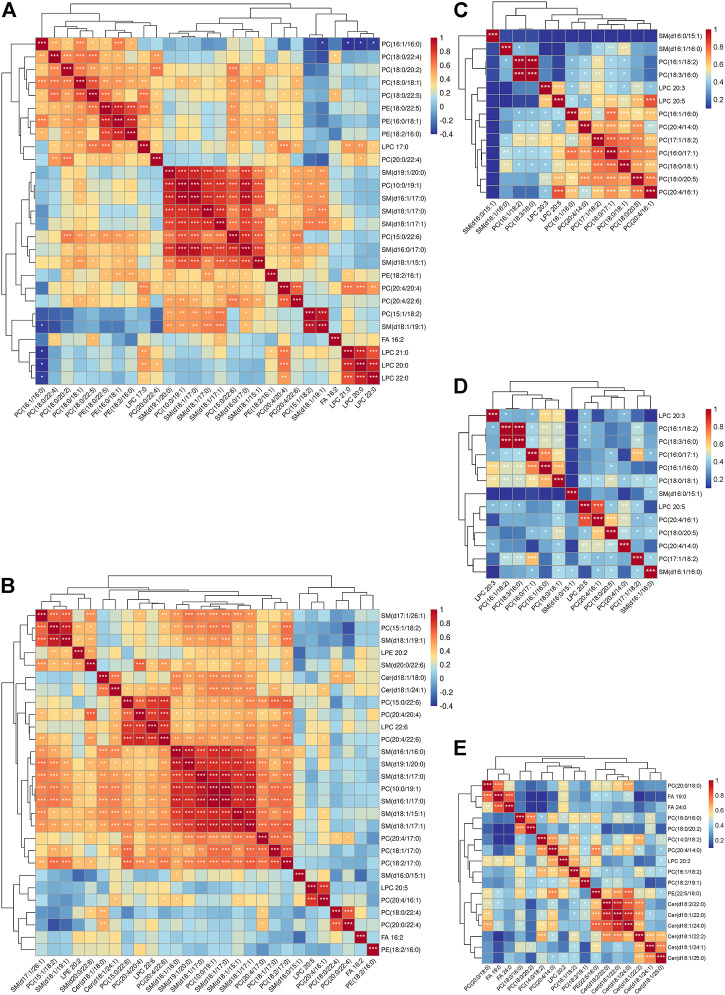
Heatmaps illustrating Spearman’s correlations between the altered metabolites. **(A)** Correlation heatmap of altered metabolites (MP vs. HC-MP) in the MP subgroup. **(B)** Correlation heatmap of altered metabolites (SP vs. HC-SP) in the SP subgroup. **(C)** Correlation heatmap of altered metabolites (MP vs. SP) in the MP subgroup. **(D)** Correlation heatmap of altered metabolites (MP vs. SP) in the SP subgroup. **(E)** Correlation heatmap of altered metabolites (PVM vs. PV) in the PVM group. Correlation analysis performed with Spearman’s correlation coefficient, *p < 0.05, **p < 0.01, and ***p < 0.001. HC-MP, healthy controls matched to the group with mild psoriasis vulgaris; HC-SP, healthy controls matched to the group with severe psoriasis vulgaris; MP, mild psoriasis vulgaris; SP, severe psoriasis vulgaris; PV, psoriasis vulgaris without metabolic diseases; PVM, psoriasis vulgaris with metabolic diseases.

**FIGURE 4:**
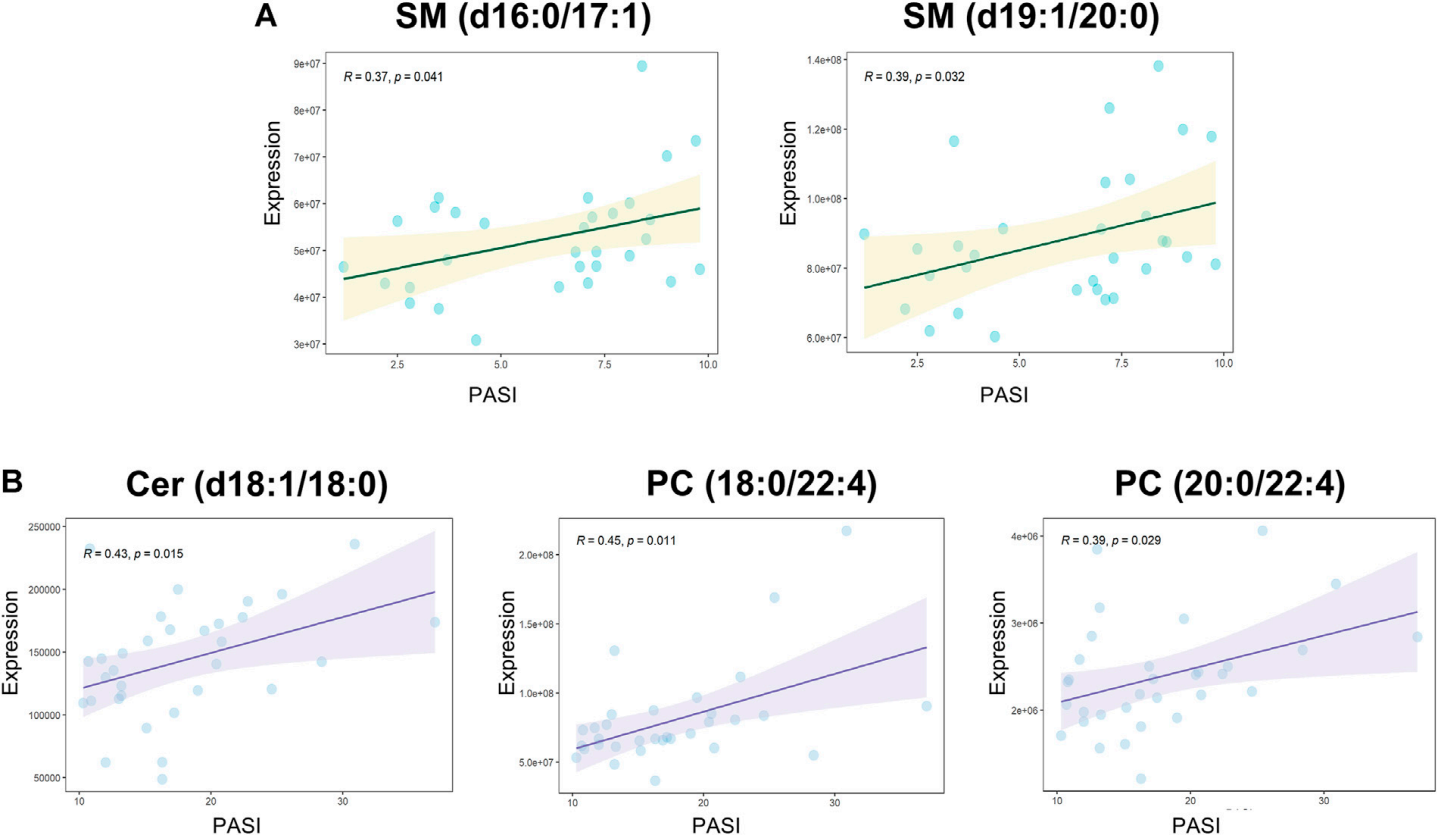
Spearman’s correlations between altered metabolites and the PASI. **(A)** Correlation analyses between PASI and altered metabolites (MP vs. HC-MP) in the MP subgroup: SM (d16:1/17:0): *R* = 0.37, *p* = 0.041; SM (d19:1/20:0): *R* = 0.39, *p* = 0.032. **(B)** Positive correlations between PASI and altered metabolites (SP vs. HC-SP) in the SP subgroup: Cer (d18:1/18:0): *R* = 0.42, *p* = 0.015; PC (18:0/22:4): *R* = 0.45, *p* = 0.01; and PC (20:0/22:4): *R* = 0.39, *p* = 0.029. PASI, Psoriasis Area and Severity Index; HC-MP, healthy controls matched to the group with mild psoriasis vulgaris; HC-SP, healthy controls matched to the group with severe psoriasis vulgaris; MP, mild psoriasis vulgaris; SP, severe psoriasis vulgaris.

Spearman’s correlation was applied to explore the associations between all the measured metabolites and the patient’s PASI scores (Supplementary Table S4). SM (d16:1/16:1) and LPE 20:4 were negatively correlated with PASI, whereas a positive correlation was observed between Cer (d18:1/18:0) and the PASI. The correlation analysis between all the measured metabolites and biochemical indicators including cholesterol, triglyceride, high-density lipoprotein cholesterol (HDL-C), and low-density lipoprotein cholesterol (LDL-C) was also performed to enrich the significance of the harvested lipid biomarkers (Supplementary Table S5). Surprisingly, Cer (d18:1/18:0) and SM (d16:1/16:1) were not only correlated with PASI but also strongly correlated with biochemical indicators. Cer (d18:1/18:0) was strongly positively correlated with cholesterol, triglyceride, and LDL-C, whereas SM (d16:1/16:1) had the strongly positive correlations with cholesterol, HDL-C, and LDL-C. Furthermore, we observed positive correlations (all values of *p* < 0.05) among most of the altered metabolites (Figure 3). SM and PC, and PC and PE showed a strong correlation within the altered metabolites in the MP subgroup (Figure 3A). In the SP subgroup, a strong correlation between SM and PC, and PC and LPC was observed in the altered metabolites (Figure 3B). This observation indicates that the progression of PV symptoms may be associated with an alteration of lipid metabolism. Moreover, we also found a strong correlation between PC and FFA, and PE and Cer in the PVM group (Figure 3E). To further investigate the severity biomarkers of PV, we conducted Spearman’s correlation analysis between the PASI scores and the altered metabolites (MP vs. HC-MP and SP vs. HC-SP). The results show that SM (d19:1/20:0) and SM (d16:1/17:0) in the MP subgroup were positively correlated with the PASI scores (Figure 4A). In addition, PC (18:0/22:4), PC (20:0/22:4), and Cer (d18:1/18:0) in the SP subgroup were positively correlated with the PASI scores (Figure 4B). There was no metabolite that strongly was correlated with the PASI in both the MP and the SP subgroups.

## Discussion

**FIGURE 5:**
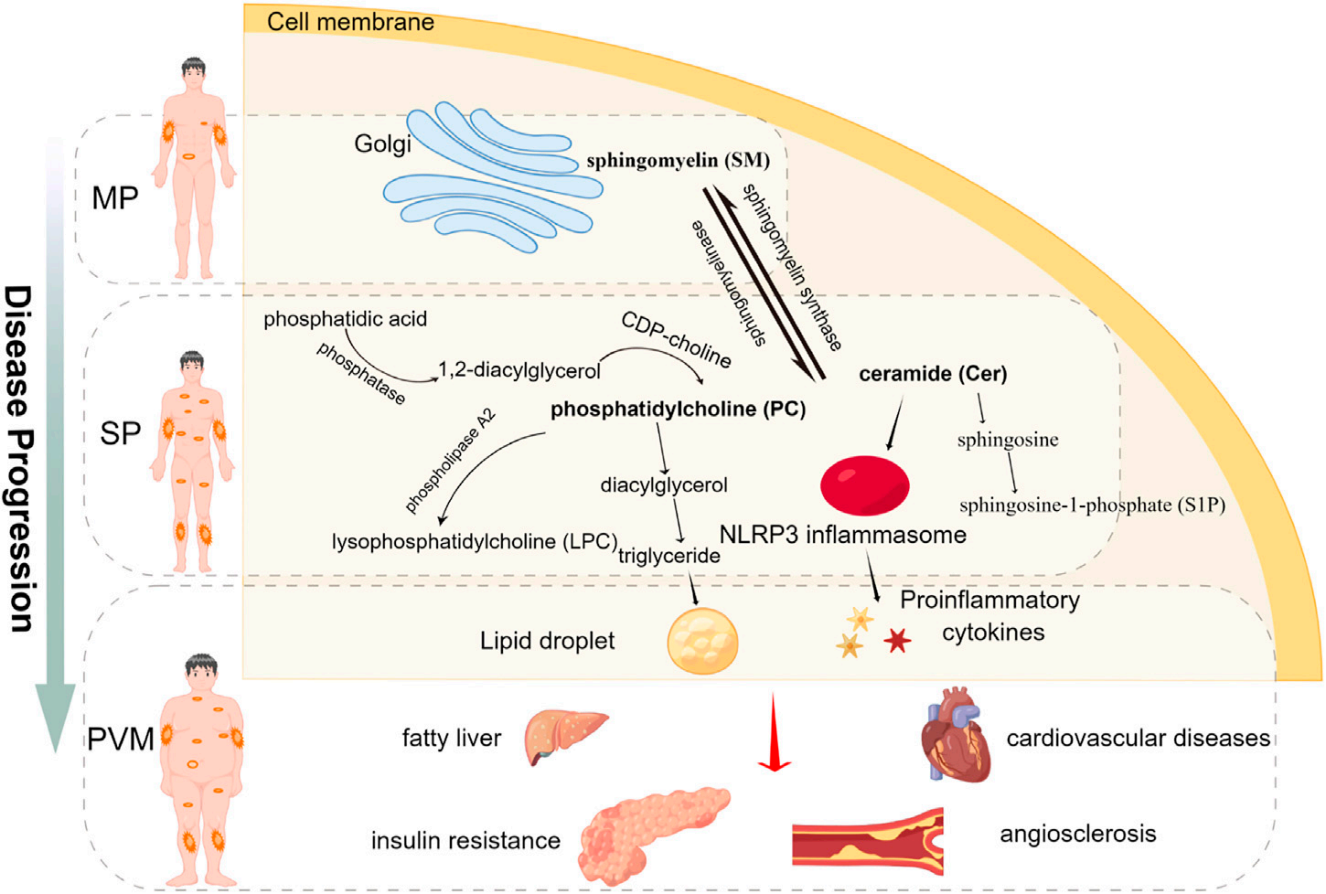
Graphical abstract showing the major findings of the study.

Based on the idea that subtyping of PV may contribute to the development of personalized treatment, we explored the molecular evidence for alterations in lipid metabolism at different severity levels of the disease using serum metabolomics. The identified metabolic disorders related to the progress of PV are summarized in Figure 5 excessive hydrolysis causes the accumulation of PC and subsequently lipids droplet formates , which may be an important factor that contributes to disease progression. Using the targeted UPLC-MS/MS-based lipidomics platform to characterize the serum samples of patients, we inspected the pathological mechanism underlying PV that is closely associated with complex lipid metabolism dysfunction and thereby identified a set of PV’s lipid biomarkers. The correlation analysis indicated that Cer (d18:1/18:0) and SM (d16:1/16:1) were not only correlated with PASI but also has strongly positive correlations with biochemical indicators in our analysis. Positive correlations were observed among the most altered metabolites. Among the altered metabolites in the MP subgroup, SM and PC levels showed a strong correlation. A strong correlation between SM and PC, and PC and LPC was observed for the altered metabolites in the SP subgroup. We focused on the biomarkers associated with the severity of PV and found that in the MP subgroup, SM (d16:0/17:1) and SM (d19:1/20:0) were positively correlated with the PASI, whereas in the SP subgroup, PC (18:0/22:4), PC (20:0/22:4), and Cer (d18:1/18:0) were positively correlated with PASI scores. These results suggest that SM disorders dominated the lipid abnormalities in the MP subgroup, while Cer and PC disorders were predominant in the SP subgroup. In addition, we found more patients with severe PV complicated by metabolic diseases than patients with mild PV, suggesting that dysfunction in lipid metabolism may contribute to the progression of PV. Cer, FFA, PC, LPC, and PE levels were significantly higher in patients with PV complicated by metabolic diseases than in those without metabolic diseases. Although the pathogenesis of metabolic complications of PV is unclear, it has been linked to lipid accumulation caused by dysfunctional lipid metabolism. Our findings thus offer novel insights into the metabolic nature of PV. The metabolic biomarkers we suggest for the subtyping of PV may assist practitioners in identifying potentially important risk factors in the future and support the development of precision medicine treatment for PV.

SM is primarily produced in the Golgi apparatus and transferred to all other cellular membranes (Campelo et al., 2017). Sphingomyelinase catalyzes the hydrolysis of the phosphodiester bond of sphingomyelin and yields Cer and PC after stimulation by TNF-α or IL-1β (Sindhu et al., 2021). In our sample, SM disorders dominated the lipid abnormalities observed in the MP subgroup and thus provide possible treatment targets for MP. Our results indicate that SM was reduced in patients in the MP subgroup compared to HCs. Among all the measured lipid metabolites, SM (d16:1/16:1) is negatively correlated with PASI and had strongly positive correlations with cholesterol, HDL-C, and LDL-C. SMs are the most abundant surface components of plasma lipoproteins including LDL and HDL, and abnormal SM metabolism dysfunction impairs the structure and biofunctions of LDL and HDL (Iqbal et al., 2017; Tsuji et al., 2021). Reduced SM levels may contribute to disease progression, as it has been reported that SM supplements appear to protect against aberrant lipid metabolism, intestinal dysbiosis, and inflammation (Norris et al., 2017; Norris et al., 2019). The SM derivative sphingosine-1-phosphate (S1P) activates differentiation and inhibits keratinocyte proliferation (Jeon et al., 2020). S1P receptor agonists have been found to alleviate psoriasis-like dermatitis in mice (Liu et al., 2021). Moreover, controversially, a high-fat diet can induce sphingomyelin accumulation in the liver (Chocian et al., 2010). The lack of sphingomyelinase promotes sphingomyelin accumulation and causes defective cholesterol trafficking and efflux, which may occur in diabetes and atherosclerotic heart disease (Leventhal et al., 2001; Summers, 2006). The seemingly opposite functions of SM may thus explain why SM is deficient in MP but positively correlated with the PASI.

Cer and PC were found to be related to lipid metabolism dysfunction in the SP subgroup. Cer is the key precursors for the synthesis of other sphingolipids, and the decreased SM and elevated Cer values suggest excessive hydrolysis or impaired synthesis of SM in this group. Different Cer species have specific and sometimes opposing biological functions (Gomez-Larrauri et al., 2020). Cer promotes the NLRP3 inflammasome, which increases IL-1β levels (Vandanmagsar et al., 2011) and enhances TNF-α, MCP-1, and IL-6 production in adipocytes (Samad et al., 2006). High concentrations of Cer in the white adipose tissue exacerbate chronic inflammation and insulin resistance (Kolak et al., 2007; Chaurasia et al., 2016), which is an independent risk factor for cardiovascular death in patients with stable coronary artery disease (Laaksonen et al., 2016). However, the overall serum content of Cer was reported to be lower in psoriatic patients (Myśliwiec et al., 2017), which is not in line with our results. Cer deficiency in the stratum corneum has also been found to lead to the dry desquamation of the skin (Bocheńska and Gabig-Cimińska, 2020). The correlation analysis highlighted that the expression of Cer (18:1/18:0) was strongly positively correlated with PASI not only in an early stage of PV such as SP group but also in all enrolled patients with PV, which can be considered a severity biomarker for clinical reference.

PC plays a role in regulating blood lipoprotein homeostasis. Impaired hepatic PC biosynthesis significantly reduces the concentrations of circulating very low-density lipoproteins as well as high-density lipoproteins (HDLs) (da Silva et al., 2020; Sprenger et al., 2021). HDL not only eliminates excess cholesterol but also has anti-inflammatory and antioxidant properties (Kuai et al., 2016). The phospholipid composition determines HDL function and secretion. The composition of HDL phosphatidylcholine affects the hepatic absorption of HDL lipid cargo (Kadowaki et al., 1993). In our sample, some kinds of PC were downregulated, while some were upregulated in patients with PV serum, which is consistent with the results of previous studies (Zeng et al., 2017; Li et al., 2020). Downregulated PC was also found in patients with PV (Łuczaj et al., 2020), potentially due to the excessive proliferation of keratinocytes during the progression of PV. Dietary PC supplementation substantially ameliorates lipid transportation dysfunction and atherosclerosis (Aldana-Hernández et al., 2021). Excess PC may be catabolized and promote triglyceride synthesis and triglyceride-mediated steatosis (Martínez-Uña et al., 2013), which increases the risk of obesity-related diseases in patients with PV. The positive correlation between triglyceride and PC in our experiment also supported this conclusion. The significantly high triglyceride levels observed in our comparison of the PV and PVM groups may have been a result of PC accumulation. PC disorders dominated the lipid abnormalities observed in the SP subgroup, which suggests the need for attention to lipoprotein functions when it comes to the development of targeted preventive treatment for PV.

LPC is generated from PC through the hydrolysis of phospholipase A2 and is the core component of oxidatively damaged low-density lipoprotein (Shoda et al., 1997; Ohigashi et al., 2019). Thus, the altered PC and LPC levels we observed in the SP subgroup were strongly correlated. Increasing evidence indicates that LPC levels are elevated in inflammation-related diseases, including psoriasis (Zeng et al., 2017; Li et al., 2020). Nevertheless, in the current study, the entire PC composition was decreased. LPC usually has a bidirectional effect on inflammation regulation, resulting in various functions in the progression of inflammatory diseases (Liu et al., 2020). LPC with polyunsaturated acyls can curtail inflammation induced by saturated LPC by acting as an anti-inflammatory lipid mediator (Hung et al., 2012). Moreover, LPC can reinforce the immunosuppressive function of regulatory T cells by increasing the production of TGF-β and Foxp3 via the G2A-JNK pathway (Hasegawa et al., 2011).

The top three metabolic comorbidities in the PVM group were hyperlipidemia (48%), hypercholesterolemia (28%), and hyperlipidemia and hypertension (12%), which were presented with serum accumulation of Cer, PC, LPC, FFA, and PE. Elevated PC levels promote triglyceride synthesis and triglyceride-mediated steatosis (Łuczaj et al., 2020). In addition to the routine synthesis pathway (CDP-choline pathway) (Kennedy and Weiss, 1956), the liver possesses a unique PC synthesis pathway that involves three consecutive methylations of the ethanolamine moiety of PE catalyzed by PE methyltransferase and accounts for approximately 30% of hepatic PC synthesis (Sundler and Akesson, 1975). Abnormal cellular PC/PE molar ratios in diverse tissues have been associated with disease progression and alterations in energy metabolism (van der Veen et al., 2017). Ottas et al. (2017) found that PE levels are high in the serum of patients with psoriasis. This finding is consistent with our observations. Increased PE fraction breakdown could release arachidonic acid, which would be accompanied by lower quantities of bile acids in the serum of patients with psoriasis, as well as poorer antioxidant indicators, such as glutathione (Sorokin et al., 2018; Miura et al., 2021). We also found higher Cer levels in patients with only PV than HCs, but their levels were still lower than those of patients with PV and metabolic diseases, suggesting that Cer may contribute to metabolic and inflammatory responses. Circulating FFAs are the primary energy source for almost all tissues and are obtained mostly from adipose tissue lipolysis, and elevated saturated fatty acids levels exacerbate early psoriatic skin inflammation (Herbert et al., 2018). Fatty acid synthesis appears to drive the formation of Th17 cells (Young et al., 2017; Nicholas et al., 2019). Polyunsaturated fatty acids can induce thrombosis and proinflammation, resulting in an increased prevalence of atherosclerosis, obesity, and diabetes (Simopoulos, 2013; Kromhout and de Goede, 2014; Simopoulos, 2016).

This study has some limitations. The reported lipids could not be identified with high certainty using MS/MS because of poor lipid abundance, ion suppression, and co-elution. Moreover, the correlations obtained in the current study were retrieved from cross-sectional data; thus, the identified biomarkers need to be verified in a large independent cohort before being applied in clinical practice. Future work should focus on a detailed understanding of the intricate mechanisms governing the interaction between systemic inflammation and abnormal lipid metabolic responses.

## Conclusion

Classification into molecular subtypes enables the identification of potential therapeutic targets that are specific to complex and refractory diseases, such as PV. We validated the subtypes of PV with different lipid metabolic profiles and identified their molecular biomarkers related to disease progression. Our findings therefore contribute to the understanding of the pathogenesis and development of novel strategies for precision treatment of PV.

## Data Availability

The raw data supporting the conclusion of this article will be made available by the authors, without undue reservation.

## Abbreviation

BMI, body mass index; Cer, ceramide; CV, coefficient of variation; FFA, free fatty acid; HC, healthy control; HC-MP, healthy controls matched with mild psoriasis vulgaris; HC-SP, healthy controls matched with severe psoriasis vulgaris; HDL-C, high-density lipoprotein cholesterol; LDL-C, low-density lipoprotein cholesterol; LPC, lysophosphatidylcholine; LPE, lysophosphatidylethanolamine; MP, mild psoriasis vulgaris; PASI, Psoriasis Area and Severity Index; PC, phosphatidylcholine; PE, phosphatidylethanolamine; PLS-DA, partial least squares discriminant analysis; PV, psoriasis vulgaris; PVM, psoriasis vulgaris with metabolic diseases; QC, quality control; SM, sphingomyelin; SP, severe psoriasis vulgaris; UPLC-MS/MS, ultra-performance liquid chromatography–tandem mass spectrometry; VIP, variables of importance in projection.
