# Supplementary figures and images for: Toward Personalized Interventions for Psoriasis Vulgaris: Molecular Subtyping of Patients by Using a Metabolomics Approach
Source: Front Mol Biosci. 2022 Jul 19;9:945917. doi: 10.3389/fmolb.2022.945917 (PMC9343857; doi:10.3389/fmolb.2022.945917)

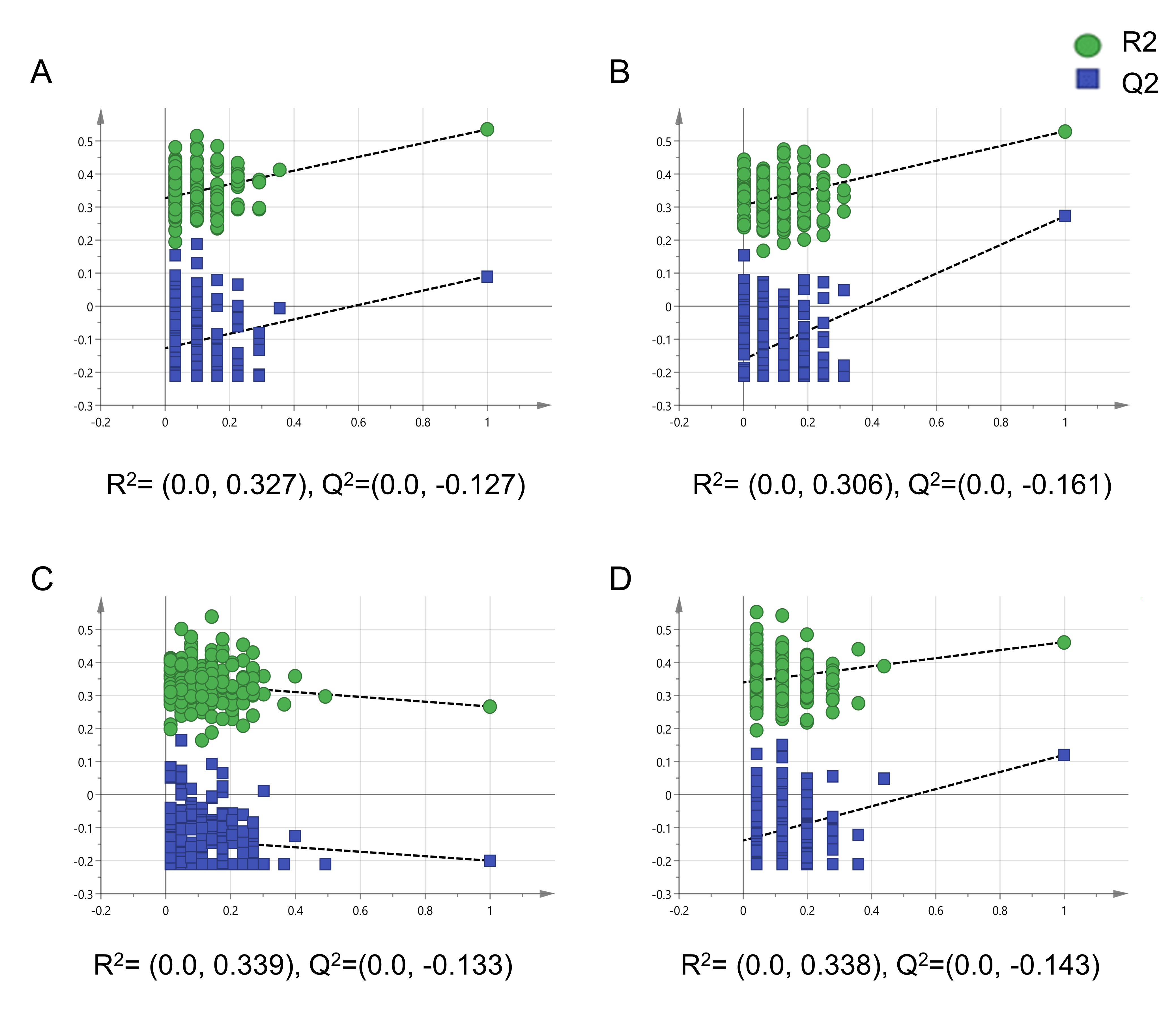

Supplement: Supplementary file 3 [file Image1.tif]
